# MicroRNA expression profiling to identify and validate reference genes for relative quantification in colorectal cancer

**DOI:** 10.1186/1471-2407-10-173

**Published:** 2010-04-29

**Authors:** Kah Hoong Chang, Pieter Mestdagh, Jo Vandesompele, Michael J Kerin, Nicola Miller

**Affiliations:** 1 Department of Surgery, National University of Ireland, Galway, Republic of Ireland; 2 Center for Medical Genetics, Ghent University Hospital, Ghent, Belgium; 3 Biogazelle, Ghent, Belgium

## Abstract

**Background:**

Advances in high-throughput technologies and bioinformatics have transformed gene expression profiling methodologies. The results of microarray experiments are often validated using reverse transcription quantitative PCR (RT-qPCR), which is the most sensitive and reproducible method to quantify gene expression. Appropriate normalisation of RT-qPCR data using stably expressed reference genes is critical to ensure accurate and reliable results. Mi(cro)RNA expression profiles have been shown to be more accurate in disease classification than mRNA expression profiles. However, few reports detailed a robust identification and validation strategy for suitable reference genes for normalisation in miRNA RT-qPCR studies.

**Methods:**

We adopt and report a systematic approach to identify the most stable reference genes for miRNA expression studies by RT-qPCR in colorectal cancer (CRC). High-throughput miRNA profiling was performed on ten pairs of CRC and normal tissues. By using the mean expression value of all expressed miRNAs, we identified the most stable candidate reference genes for subsequent validation. As such the stability of a panel of miRNAs was examined on 35 tumour and 39 normal tissues. The effects of normalisers on the relative quantity of established oncogenic (*miR-21 *and *miR-31*) and tumour suppressor (*miR-143 *and *miR-145*) target miRNAs were assessed.

**Results:**

In the array experiment, *miR-26a*, *miR-345*, *miR-425 *and *miR-454 *were identified as having expression profiles closest to the global mean. From a panel of six miRNAs (*let-7a*, *miR-16*, *miR-26a*, *miR-345*, *miR-425 *and *miR-454*) and two small nucleolar RNA genes (*RNU48 *and *Z30*), *miR-16 *and *miR-345 *were identified as the most stably expressed reference genes. The combined use of *miR-16 *and *miR-345 *to normalise expression data enabled detection of a significant dysregulation of all four target miRNAs between tumour and normal colorectal tissue.

**Conclusions:**

Our study demonstrates that the top six most stably expressed miRNAs (*let-7a*, *miR-16*, *miR-26a*, *miR-345*, *miR-425 *and *miR-454*) described herein should be validated as suitable reference genes in both high-throughput and lower throughput RT-qPCR colorectal miRNA studies.

## Background

Mi(cro)RNAs are short RNA molecules that bind (generally) to 3' UTR sequences of target messenger RNAs (mRNAs), thereby modulating their expression patterns. This modulated gene expression is manifest either as translational repression [1], or mRNA degradation whereby the RNA interference pathway is initiated to remove targeted sequences [2]. MiRNAs play major roles in governing diverse biological processes such as differentiation, proliferation, and apoptosis [3,4]. Individual miRNAs have been ascribed oncogenic and tumour suppressor functions [5], and aberrant miRNA expression has been implicated in many malignancies, including colorectal cancer (CRC) [6,7]. Previous study demonstrated that miRNA profiles may be more accurate in disease classification than mRNA profiles [8]. Moreover, miRNAs have been associated with CRC pathogenesis [9,10], microsatellite stability status [11,12], therapeutic outcome and prognosis [12-15].

High-throughput technology such as microarrays enables simultaneous quantification of hundreds of miRNAs in a single RNA sample. Meaningful interpretation of such large datasets has been made possible by recent advances in bioinformatics. It is critical that the findings of microarray screening methodologies are validated to produce scientifically robust results, using the most sensitive and reproducible method of gene expression quantitation, reverse transcription quantitative PCR (RT-qPCR) [16]. In order to achieve accurate, reproducible and biologically relevant miRNA RT-qPCR data, non-biological sample-to-sample variation that could be introduced by protocol-dependent inconsistencies has to be corrected for by using reference genes. Use of unreliable reference genes for normalisation may lead to inaccurate quantitation of miRNAs of interest [17,18]. Previous studies have demonstrated that a single universal reference gene for all tissue types is unlikely to exist [19-23], and the use of a single reference gene for normalisation leads to large errors and is therefore inappropriate [22,24].

Despite increasing miRNA expression studies in CRC, no previous report detailed a robust identification and validation strategy for suitable reference genes for normalization. The aim of this study was to identify the most stable reference genes using a high-throughput approach, in ten pairs of stage II colorectal tumour and normal tissues. Following TaqMan array card analysis and the established approach of finding miRNAs whose expression pattern is similar to the global mean expression [25], *miR-26a*, *miR-345*, *miR-425 *and *miR-454 *were identified as the most stably expressed miRNAs. The stability of these miRNAs was further assessed by RT-qPCR in 74 colorectal tissues with an expanded panel of candidate reference miRNAs (*let-7a*, *miR-16*) and two small nucleolar RNAs (snoRNAs, *RNU48 *and *Z30*). Well established oncogenic miRNAs in CRC: *miR-21 *[7,13,26] and *miR-31 *[7], and tumour suppressor miRNAs: *miR-143 *[6,27,28] and *miR-145 *[6,7,12,27] were used as target miRNAs to determine the effect of reference gene choice on relative quantitation.

## Methods

### Colorectal tissue samples

Primary colorectal tissues consisting of 35 tumour specimens and 39 normal tissues were obtained from 40 patients undergoing surgical resection or diagnostic endoscopy at Galway University Hospital, Galway, Ireland. High-throughput miRNA profiling was performed on ten pairs of corresponding tumour and normal tissues from patients with stage II CRC [29], and these form part of the subsequent validation cohort. Tissue samples were immediately snap-frozen in liquid nitrogen following retrieval and stored at -80°C. Written informed consent was obtained from each patient and the study was granted approval by the Clinical Research Ethics Committee of Galway University Hospital. Clinicopathological data was collected prospectively and is summarised in Table 1.

**Table 1:** Clinicopathological data for 40 patients with colorectal cancer (tissues: colorectal tumour n = 35 and normal n = 39)

Characteristics (n = 40)	Number (percentage)
Age (mean ± standard deviation)	66.7 ± 13.1

Sex	
Male	28 (70.0)
Female	12 (30.0)

Location of tumors	
Colon	11 (27.5)
Rectum	29 (72.5)

Pathologic T classification	
Tx	2 (5.0)
Tis	1 (2.5)
T1	4 (10.0)
T2	9 (22.5)
T3	12 (30.0)
T4	11 (27.5)
N/A	1 (2.5)

Pathologic N classification	
Nx	2 (5.0)
N0	22 (55.0)
N1	11 (27.5)
N2	4 (10.0)
N/A	1 (2.5)

Metastasis classification	
M0	34 (87.5)
M1	6 (12.5)

AJCC classification	
Stage 0	1 (2.5)
Stage I	10 (25.0)
Stage II	10 (25.0)
Stage III	11 (27.5)
Stage IV	6 (12.5)
pCR	2 (10.0)

Differentiation	
Well	1 (2.5)
Moderate	24 (60.0)
Poor	8 (20.0)
N/A	7 (35.0)

AJCC, American Joint Committee on Cancer; pCR, pathologic complete response following neoadjuvant therapy.

### RNA extraction

To isolate small RNA (<200 nucleotides), approximately 100 mg of tissue was homogenised using a bench-top homogeniser (Polytron PT1600E, Kinematica AG, Lucerne, Switzerland) in 1-2 mL of Qiazol (Qiagen, UK). Subsequent miRNA extraction was performed using the RNeasy Mini Kit and the RNeasy MinElute Cleanup Kit (Qiagen) according to the manufacturer's instructions. Concentration and purity of miRNA was assessed using the Nanodrop 1000 spectrophotometer (Nanodrop Technologies Inc., USA). Qualitative analysis of miRNA was performed using the Agilent 2100 Bioanalyzer and the Small RNA Assay (Agilent Technologies, USA) to determine the percentage of miRNA in the small RNA fraction.

### TaqMan array cards

A TaqMan Human MicroRNA array card is a high throughput PCR-based miRNA array that enables analysis of 384 miRNA assays on a microfluidic card. Each card contains a mammalian U6 assay repeated 4 times, and an assay unrelated to any mammalian species *ath-miR-159a *to provide a process control. Simultaneous synthesis of cDNA for mature miRNAs was performed using Megaplex Reverse Transcription Human Pool A (Applied Biosystems), which is a set of pre-defined pools of 380 stem-looped reverse transcription primers. RT-qPCR was performed using the Applied Biosystems 7900HT Fast Real-Time PCR System, and default thermal-cycling conditions.

### Validation RT-qPCR

First strand cDNA was synthesised using gene-specific stem-loop primers. The primer sequences of *let-7a *and *miR-16 *have been previously described [30]. Primers were obtained from MWG Biotech (Ebersberg, Germany) if sequences were available. Otherwise, assays containing stem-looped primer were purchased from Applied Biosystems. All reagents were included in the High-Capacity cDNA Reverse Transcription Kit (Applied Biosystems). The reaction was performed using a GeneAmp PCR system 9700 thermal cycler (Applied Biosystems) with samples incubated at 16°C for 30 minutes, 42°C for 30 minutes and 85°C for 5 minutes. An RT-negative control was included in each batch of reactions.

The PCR reactions were carried out in final volumes of 20 μL using the Applied Biosystems 7900HT Fast Real-Time PCR System. Reaction mix consisted of 10 μL 2 × TaqMan Fast Universal PCR Master Mix, No AmpErase UNG, 1 μL 0.2 μM TaqMan probe, 3 μL 1.5 μM of forward primer, 1.4 μL 0.7 μM reverse primer, and 1.33 μL of cDNA. The PCR reactions were initiated with 10 minutes incubation at 95°C, followed by 40 cycles of 95°C for 15 seconds and 60°C for 60 seconds. Inter-assay control and calibrator were included in each 96-well plate. All reactions were performed in triplicate. The threshold standard deviation for intra- and inter-assay replicates was 0.3. PCR amplification efficiencies were calculated for each candidate reference gene assay using the formula E = (10^-1/slope ^- 1) × 100, using the slope of the plot of quantification cycle (Cq) versus log input of cDNA (10-fold dilution series). PCR amplification efficiencies for each candidate reference gene are shown in Table 2.

**Table 2:** Details of candidate reference genes and their amplification efficiencies

Name	Length (nt)	RNA species	Accession number	Function	E (%)
*let-7a*	22	miRNA	MI0000060*	Negatively regulates RAS oncogene [37]	100.0

*miR-16*	22	miRNA	MI0000070*	Negatively regulates B-cell lymphoma mRNA in chronic lymphocytic leukaemia [38]	100.0

*miR-26a*	22	miRNA	MI0000083*	Involved in myogenesis and osteogenic differentiation [39,40]	99.8

*miR-345*	22	miRNA	MI0000825*	Overexpressed in malignant mesothelioma [41]	100.8

*miR-425*	22	miRNA	MI0001448*	No functionally verified targets	101.2

*miR-454*	22	miRNA	MI0003820*	No functionally verified targets	101.8

*RNU48*	63	snoRNA	NR_002745**	Guides the 2'O-ribose methylation of 28S rRNA [42]	100.0

*Z30*	97	snoRNA	AJ007733**	Guides the methylation of the Am47 residue in U6 snoRNA [43]	99.4

*mirBase database accession number **Entrez gene ID

rRNA, ribosomal RNA; snoRNA, small nucleolar RNA; E, amplification efficiency.

### Data analysis

High throughput data generated from TaqMan array card RT-qPCR was analysed using qbase^PLUS ^software (Biogazelle, Belgium) [31]. Average values of triplicate Cq values were converted to relative quantities for NormFinder and geNorm analysis [21,32]. The relative expression of target miRNAs (*miR-21*, *miR-31*, *miR-143 *and *miR-145*) normalised to one or more reference candidates was also determined using qbase^PLUS ^software employing a generalised and universally applicable quantification model based on efficiency correction, error propagation and multiple reference gene normalisation [31].

Statistical analysis was performed using SPSS 14.0 (Chicago, IL, USA) and Minitab^® ^15 softwares (Minitab Ltd, Coventry, UK). Distribution of continuous data was determined using the Kolmogorov-Smirnov Z test. Two-sample *t *test was used to compare log 10 Cq values of candidate reference genes, and log 10 relative quantities of target miRNAs between tumour and normal tissues. The equivalence test was used to determine if reference genes were equivalently expressed between tumour and normal tissues [23]. Difference in variance between genes was assessed using Bartlett's test. P values of less than 0.05 were considered statistically significant for all tests.

## Results

### Identification of candidate reference genes by using the global mean expression value

We profiled a panel of 380 miRNAs and controls in 10 pairs of stage II colorectal tumour and normal tissues. To identify the most stably expressed miRNAs, a robust global mean expression normalisation strategy was applied [25]. For each individual sample, the mean Cq values of all miRNAs that were expressed, and those that were expressed below cycle 35 were calculated. Expression stability of the mean global values, the geometric means of snoRNAs (*U6*, *RNU44 *and *RNU48*) and miRNAs (*let-7a*, *miR-16*, *miR-17 *and *miR-103*) were assessed using the GeNorm algorithm. Both the geometric mean of *let-7a *and the mean global expression values for all miRNAs were found to be the most stably expressed. *U6 *RNA was the least stably expressed reference gene. Four miRNAs that showed an expression profile closest to the mean were *miR-26a*, *miR-345*, *miR-425 *and *miR-454*. Two snoRNA genes (*RNU48 *and *Z30*) and one other miRNA (*miR-16*) [17] were chosen for further validation in a larger cohort. Known functions of the candidates are listed in Table 2.

### Reference gene quantitation by RT-qPCR

RT-qPCR was performed to further evaluate the expression patterns of eight candidate reference genes in a cohort of 74 colorectal tissues. The reference genes displayed a wide range of Cq values ranging from 21.09 to 38.90. *MiR-16 *and *miR-26a *showed relatively high expression with mean Cqs of 23.61. Candidates that were moderately abundant with mean Cqs between 26 and 28 were *let-7a*, *miR-345*, *miR-425 *and *RNU48*. *Z30 *and *miR-454 *were the least expressed with mean Cqs of above 30. Among the reference genes, *miR-16 *showed the least variability while *Z30 *displayed the most. Mean and range of Cq values for all reference genes and target miRNAs are shown in Table 3.

**Table 3:** Quantification cycle (Cq) values of candidate reference genes and target miRNAs (in triplicate) in colorectal tissues

Reference gene	Cq Range	Cq Min	Cq Max	**Mean Cq ± s.d**.
*let-7a*	8.02	24.25	32.27	28.31 ± 1.60

*miR-16*	4.75	21.85	26.60	23.61 ± 1.00

*miR-26a*	6.96	21.09	28.05	23.61 ± 1.49

*miR-345*	9.37	25.14	34.51	27.43 ± 1.35

*miR-425*	10.01	24.65	34.66	27.53 ± 1.36

*miR-454*	9.64	27.76	37.40	30.14 ± 1.75

*RNU48*	9.71	22.08	31.79	26.34 ± 2.09

*Z30*	12.58	26.32	38.90	31.17 ± 2.83

**Target miRNA**				

*miR-21*	14.21	19.57	33.78	24.82 ± 4.07

*miR-31*	18.12	20.81	38.93	29.36 ± 3.75

*miR-143*	12.19	15.95	28.14	23.01 ± 2.84

*miR-145*	14.03	18.06	32.09	24.84 ± 2.79

**Figure 1:**
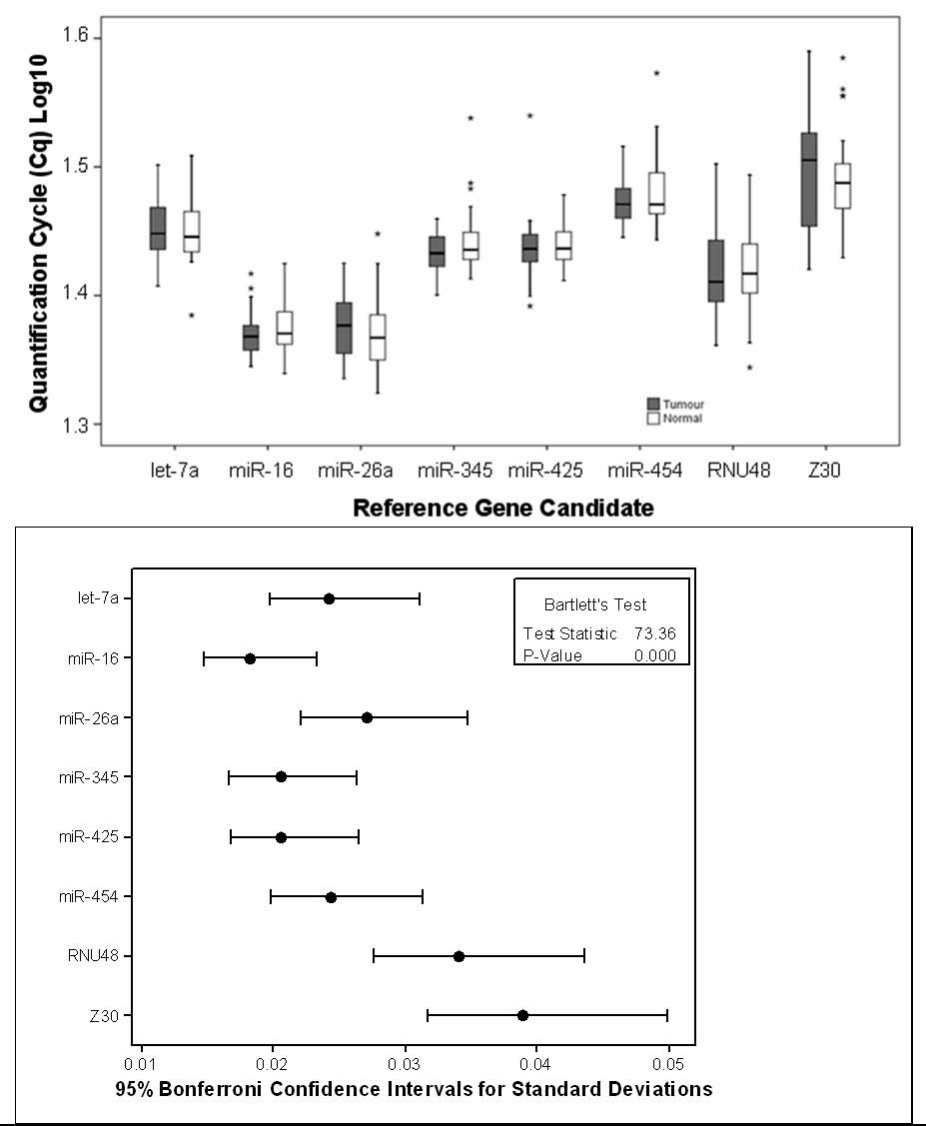
**Relative quantity and variation associated with each candidate reference gene**. (a) Quantity of candidate reference miRNAs in colorectal tumour (n = 35) and normal (n = 39) tissues as expressed as quantification cycle (Cq) values. Boxplots depict median lines, interquartile-range boxes and outliers (*). Error bars represent range of values. No significant difference (*p *> 0.05, *t*-test) was found within all reference genes between tumour and normal tissues, thus supporting further evaluation of these genes as references. (b) Variation associated with each candidate reference gene. There was a significant difference in variance (*p *< 0.001, Bartlett's test) associated with each reference gene indicating differing stabilities. *RNU48 *and *Z30 *showed greater variance than *miR-16*, *miR-345 *and *miR-425*.

**Figure 2:**
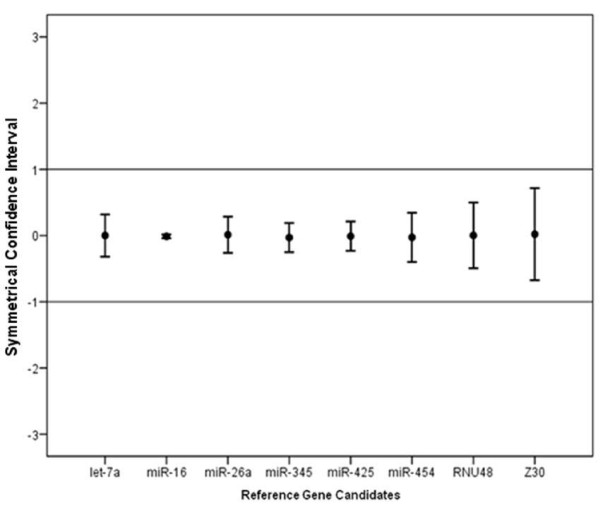
**Equivalence test for candidate reference genes**. Each line indicates the difference in logarithmic (base 2) expression level between tumour and normal tissues, with the upper and lower bars representing the upper and lower limits of symmetrical confidence intervals respectively. All genes were equivalently expressed with confidence intervals within fold change of 2 (deviation area 1, -1).

Using the Cq values of each reference gene, there was no evidence for differential expression of all of the candidate reference genes between tumour and normal tissues (*p *> 0.05; Figure 1a), thus supporting further evaluation of these candidate reference genes. A significant difference in variance between reference genes (*p *< 0.001; Figure 1b) indicated differing stabilities of these candidates. The equivalence test confirmed that all reference genes were equivalently expressed between tumour and normal colorectal tissues (Figure 2). Logarithmised relative expression values of candidate reference genes were calculated and expressed as means and matching symmetrical confidence intervals. Confidence intervals of between -1 and 1 correspond with fold change of ≤ 2, whereas confidence intervals of between -1.58 and 1.58 correspond with fold change of ≤ 3. A fold change cut-off of 3 was used as previously demonstrated [23]. The upper border of the confidence interval of > 1.58 indicates higher expression of a gene in tumours; whereas the lower border of the confidence interval of < -1.58 indicates higher expression of a gene in normal tissues.

### Analysis of reference genes' expression stability

**Figure 3:**
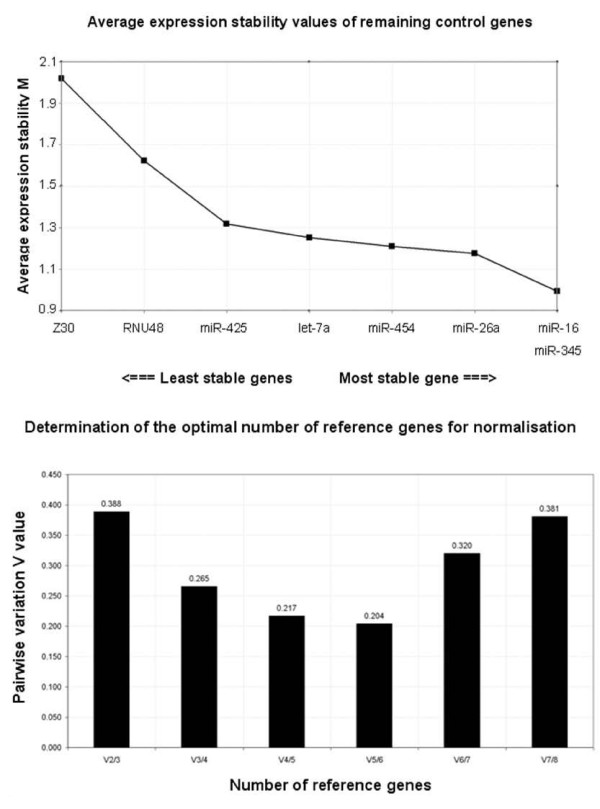
**GeNorm analysis of candidate reference genes**. (a) Ranking of candidate reference genes according to average expression stability. The least stable genes with the highest stability measure, M were excluded in a stepwise manner until the two most stable genes remained: *miR-16 *and *miR-345*. (b) Determination of optimal number of reference genes for normalisation. The GeNorm programme calculates a normalisation factor (NF) which is used to determine the optimal number of reference genes required for accurate normalisation. This factor is calculated using the variable V as the pairwise variation (Vn/Vn + 1) between two sequential NFs (NFn and NFn + 1). The number of reference genes is deemed optimal when the V value achieves the lowest, at which point it is unnecessary to include additional genes in the normalisation strategy. In this instance, the GeNorm output file indicated that optimal normalisation of gene expression could be achieved using the top five most stable reference genes.

Variable stability of reference genes was further assessed using two algorithms: NormFinder [32] and geNorm [22]. The ranking of genes as determined by these programs is summarised in Table 4. Lower stability values characterise greater gene stability. GeNorm generates a gene stability value (M) based on the average pairwise variation between all tested genes accompanied by stepwise exclusion of the least stable gene (Figure 3a). It also generates V values (V) which define the pairwise variation between two sequential normalisation factors to determine the optimal number of reference genes for normalisation (Figure 3b). NormFinder and geNorm identified *miR-345 *and *miR-16 *respectively as the most stably expressed reference genes. Consistent with the results from the TaqMan array card using the mean expression values, *let-7a*, *miR-26a*, *miR-345*, *miR-425 *and *miR-454 *were identified as five of the six most stably expressed genes in the validation dataset. Both programs selected *miR-16 *and *miR-345 *as the most stable pair of reference genes. GeNorm recommended the use of five of the six most stable genes for optimal normalisation, in line with previous reports that indicate intrinsic higher variability in cancer biopsies. Interestingly, miRNAs identified to be the most stably expressed in stage II tumour and normal tissues using high-throughput methodology remained consistently stably expressed in a larger tissue cohort consisting of tissues of varying stages.

**Table 4:** Ranking and best combination of candidate reference genes based on expression stability values calculated by NormFinder and geNorm programs

Rank	NormFinder		geNorm	
	
	Gene	Stability	Gene	Stability (M)
Best combination	*miR-16/miR-345*	0.003	*miR-16/miR-345*	0.994

1	*miR-345*	0.004	*miR-16*	1.647

2	*miR-16, miR-425*	0.005	*miR-26a*	1.693

3	*miR-454*	0.006	*miR-345*	1.697

4	*miR-26a*	0.007	*miR-425*	1.780

5	*let-7a*	0.008	*miR-454*	1.845

6	*RNU48*	0.012	*let-7a*	1.917

7	*Z30*	0.016	*RNU48*	2.365

8			*Z30*	3.212

### Effect of reference genes on relative expression of target miRNAs

**Figure 4:**
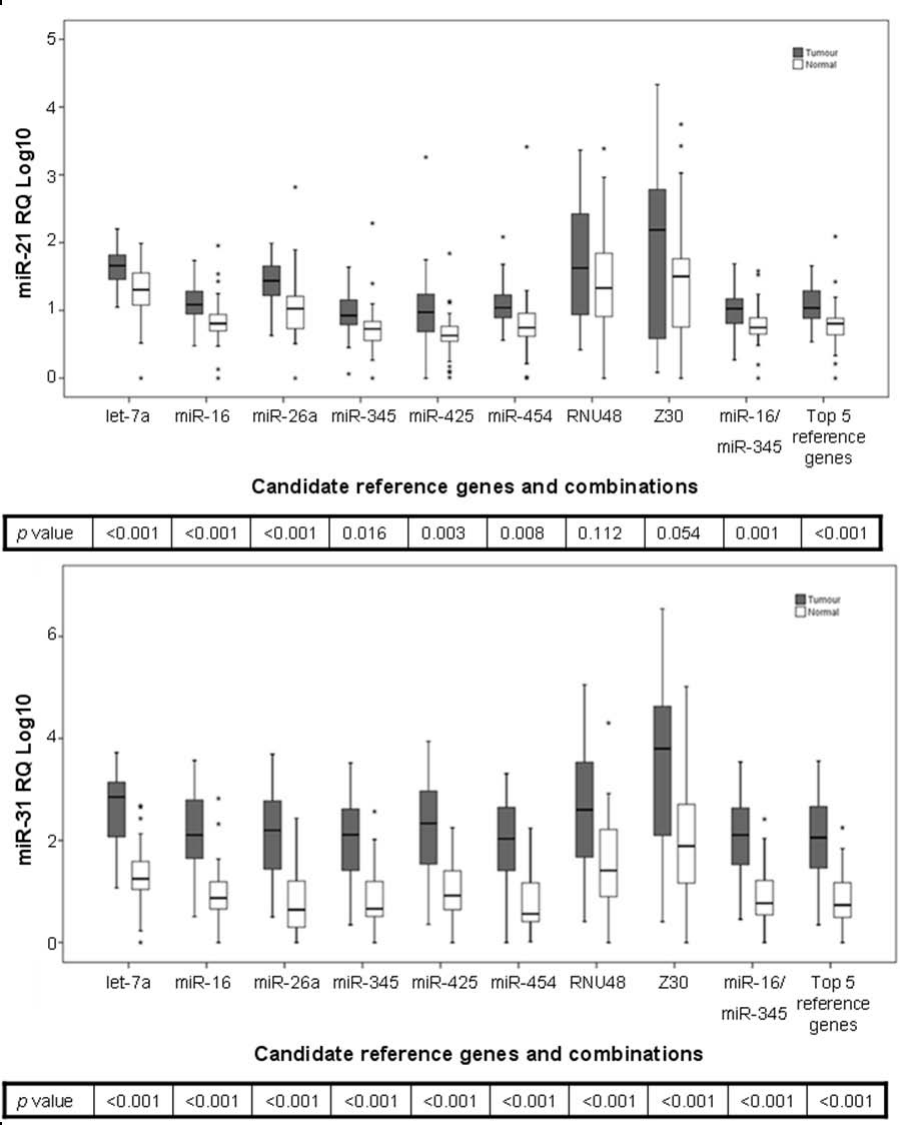
**Effect of reference gene choice on relative expression of oncogenic target miRNAs in colorectal tumour (n = 35) and normal (n = 39) tissues**. Boxplots depict median lines, interquartile-range boxes and outliers (*). Error bar represent range of values. Relative expression of oncogenic miRNAs: (a) *miR-21 *and (b) *miR-31 *between colorectal tumour and normal tissues normalised to different reference genes with *p *values indicated. The use of the two most stable reference genes: *miR-16 *and *miR-345 *detected significant dysregulation both target miRNAs between colorectal tumour and normal tissues. Dysregulation of *miR-31 *was observed regardless of the choice of reference indicating that it's highly differentially expressed in colorectal cancer.

**Figure 5:**
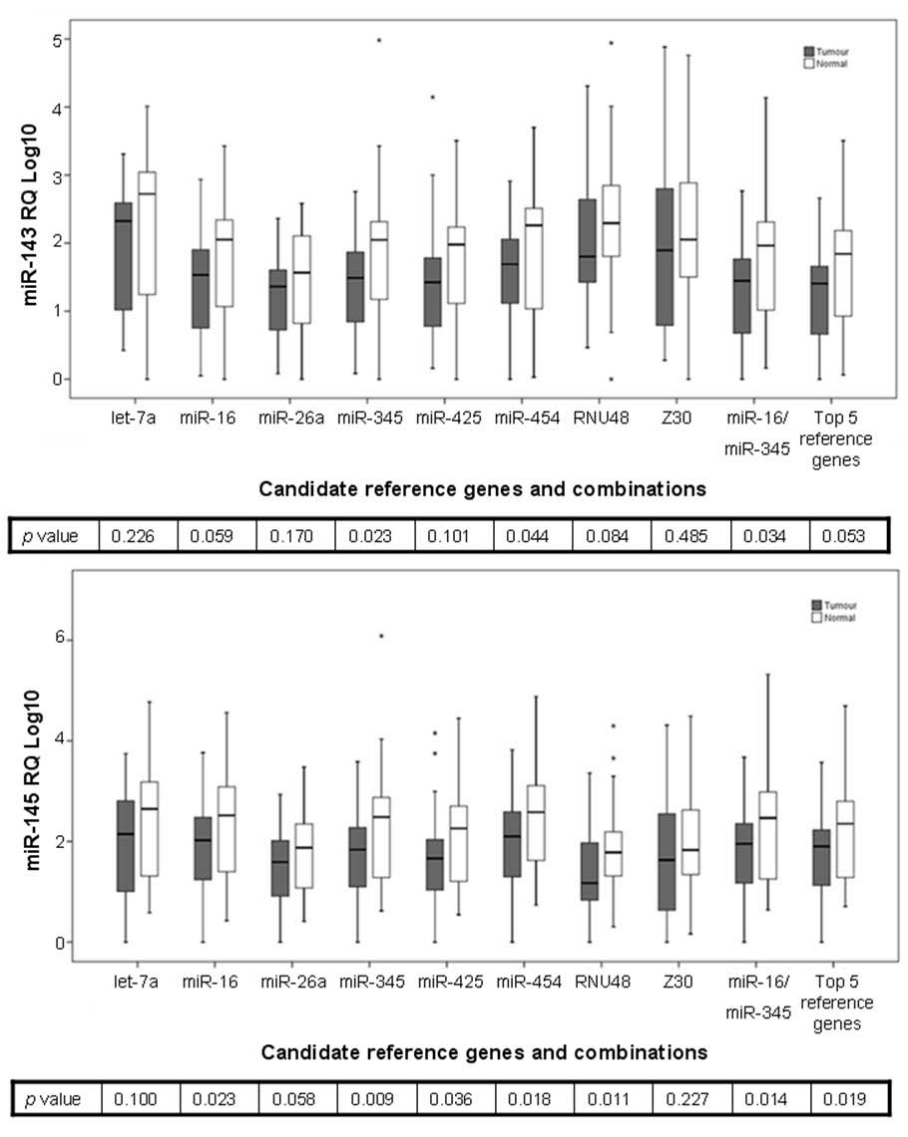
**Effect of reference gene choice on relative expression of tumour-suppressor target miRNAs in colorectal tumour (n = 35) and normal (n = 39) tissues**. Boxplots depict median lines, interquartile-range boxes and outliers (*). Error bar represent range of values. Relative expression of tumour-suppressor miRNAs: (a) *miR-143 *and (b) *miR-145 *between colorectal tumour and normal tissues normalised to different reference genes with *p *values indicated. The use of the two most stable reference genes: *miR-16 *and *miR-345 *detected significant dysregulation of both miRNAs between colorectal tumour and normal tissues.

Of the four target miRNAs evaluated after normalisation to different references, the choice of reference gene did not influence the relative quantity of *miR-31 *(*p *< 0.001) between tumour and normal tissues suggesting a highly significant differential expression of *miR-31 *in CRC (Figure 4b). Relative quantities of target miRNAs in tumour and normal tissues using different normalisers are shown in Figure 4 and Figure 5 with *p *values indicated in the box. When a single gene was used as a reference gene, only *miR-345 *and *miR-454 *detected significant difference between tumour and normal tissues in all four miRNAs. The combination of *miR-16 *and *miR-345 *detected significant up-regulation of *miR-21 *(*p *= 0.001), *miR-31 *(*p *< 0.001), and down-regulation of *miR-143 *(*p *= 0.034) and *miR-145 *(*p *= 0.014). These results highlight the importance of selecting appropriate and validated reference genes. Despite the large sample size, true biological differences in gene expression were not detected when using less stable reference genes for normalisation.

## Discussion

The discovery of miRNAs as crucial regulators of gene expression has resulted in the rapid expansion of understanding of gene regulation in normal development and disease. Previously, it was demonstrated that miRNA expression profiles may be more accurate in disease classification than mRNA expression profiles [8]. However, accurate and reliable interpretation of RT-qPCR results depends heavily on the use of suitable reference genes for normalisation to eliminate or minimise non-biological variation between test samples. While reference genes for mRNA RT-qPCR studies have been well-established, few miRNA RT-qPCR studies have detailed the validation of reference genes for normalisation to date. Rigorous normalisation of miRNA data may be more critical than that of other RNA functional classes [18]. Indeed, their capability to regulate multiple gene targets within the same pathway may amplify their biological effects [33], hence small changes in miRNA expression may be biologically and clinically significant.

Davoren et al. reported the first systematic assessments of candidate reference genes for miRNA RT-qPCR analysis in breast cancer [17]. To our knowledge, such assessment and validation of reference genes for CRC studies has not been reported. The two most commonly used normalisers *U6 *and *5S *RNAs were shown to be the two least stable RNA species [18]. The use of rRNAs as reference genes has been debated as they can be expressed at much greater levels than target RNAs resulting in difficulty quantitating a lowly expressed target RNA [20,22]. Furthermore, rRNAs have been shown to be involved in apoptosis [34] and cancer [35]. Lastly, it has been argued before that it's best to normalise genes with reference genes belonging to the same RNA class [22]. *Let-7a *was used as a normaliser in CRC miRNA RT-qPCR studies [7,10]. However, its tumour-suppressor role in CRC has been reported [27]. In a previous study, *miR-191 *and *miR-25 *were identified as the most stable pair of normalisers across 13 distinct human tissue types including 5 pairs of colon tumour and adjacent normal tissues. However, when analysis was performed on an extended cohort of lung cancer and normal tissues, *miR-17-5p *and *miR-24 *were the best normalisers [18]. This demonstrates the importance of validating suitable reference genes in a tissue-specific context. Suitable reference genes for colorectal tissue-specific studies needs to be further assessed as previous reports have demonstrated that a single universal reference gene for all tissue types is unlikely to exist [19-23].

This is the first report detailing identification and validation of suitable reference genes for normalisation of miRNA RT-qPCR in human colorectal tissues. We profiled the expression of 380 miRNAs (including *U6 *rRNA) on 20 colorectal tissues. A robust method using the mean expression value was used to identify the most stably expressed miRNAs: *let-7a*, *miR-26a*, *miR-345*, *miR-425 *and *miR-454*. Mean normalisation was previously shown to outperform other methods of normalisation in terms of better reduction of technical variation and more accurate appreciation of biological changes [25]. Validation by RT-qPCR was subsequently carried out in a larger cohort of 74 tissues with assessment of three more candidate reference genes (*miR-16*, *RNU48 *and *Z30*) [17]. Our initial validation step confirmed no difference in reference gene quantities between tumour and normal tissues, allowing subsequent use of NormFinder and geNorm as these models assume that reference genes are not differentially expressed between experimental groups. Equivalent expression of reference genes between tumour and normal tissues was then confirmed using a fold change cut-off of 3 [23]. Both NormFinder and geNorm identified *miR-16 *and *miR-345 *as the most stable normalisers. The five most stably expressed miRNAs in the TaqMan array card dataset of stage II tumours remained stably expressed when a larger cohort of variable disease stages was evaluated. This suggests that true reference genes are non-functional in the disease process, and should remain stably expressed throughout all stages, grades and subtypes.

As evident from our results, inappropriate use of reference genes can significantly alter the results of target miRNAs quantitation. With the use of the best combination of reference genes (*miR-16 *and *miR-345*), significant dysregulation of all four target miRNAs (*miR-21*, *miR-31*, *miR-143 *and *miR-145*) was detected. These target miRNAs have repeatedly been shown to be dysregulated in CRC in previous studies. However, despite a relatively large sample size, when inappropriate reference genes were used for normalisation, a true biological difference in expression between tumour and normal was not detected. Even though *miR-345 *and *miR-454 *detected significant difference between tumour and normal tissues when used alone as a reference gene, geNorm analysis identified them as only the third and the fifth most stably expressed genes. The *p *values of the differential expression of the four target miRNAs between tumour and normal tissues were slightly lower when using the *miR-16*/*miR-345 *combination in most instances, which could prove significant in a small scale study. Furthermore, previous studies have reported that the use of more than one reference genes increases the accuracy of quantitation compared to the use of a single reference gene [22,32].

## Conclusions

The results of our study have important implications for CRC translational research. The clinical and pathologically diverse nature of the tissues used in this study should be of interest to a broad spectrum of the CRC research community. While it may not be feasible due to cost and sample availability, the stability of the top six most stably expressed miRNAs in colorectal tissues (*let-7a*, *miR-16*, *miR-26a*, *miR-345*, *miR-425 *and *miR454*) should be assessed to determine the most appropriate normalisers within each study as patient and tumour characteristics may vary between different study cohorts. Furthermore, with evidence to suggest that miRNA expression in formalin-fixed paraffin-embedded (FFPE) tissue samples remains relatively stable and consistent with that in fresh-frozen samples [36], and that reference miRNA stabilities are extremely consistent between the two tissue sources procured and processed independently of one another [18], the reference genes identified in this study may be useful for miRNA RT-qPCR study in FFPE colorectal tissues. This study also demonstrated that the use of the mean expression value is a useful means of identifying stable reference genes in high-throughput miRNA profiling studies, and the findings were confirmed to be robust after external validation.

## Competing interests

The authors declare that they have no competing interests.

## Authors' contributions

KHC carried out colorectal tissue acquisition, TaqMan array card experiments, RT-qPCR assays, statistical analysis and drafted the manuscript. JV and PM were responsible for high-throughput TaqMan array card data analysis and identification of candidate reference genes using the mean expression value strategy. NM conceived, designed, supervised the study and helped to draft the manuscript. MJK participated throughout the study and critically reviewed the manuscript. All authors read and approved the final manuscript.

## Pre-publication history

The pre-publication history for this paper can be accessed here:

http://www.biomedcentral.com/1471-2407/10/173/prepub
